# ***α***2 Integrin-Dependent Suppression of Pancreatic Adenocarcinoma Cell Invasion Involves Ectodomain Regulation of Kallikrein-Related Peptidase-5

**DOI:** 10.1155/2011/365651

**Published:** 2011-12-13

**Authors:** Chia-Yao Lee, David Marzan, Grace Lin, Steve Goodison, Steve Silletti

**Affiliations:** ^1^Moores Cancer Center, University of California, San Diego, La Jolla, CA 92093, USA; ^2^Department of Pathology, University of California, San Diego, La Jolla, CA 92093, USA; ^3^Cancer Research Institute, M.D. Anderson Cancer Center, Orlando, FL 32827, USA

## Abstract

Previous reports demonstrate that the *α*2-integrin (*α*2) mediates pancreatic ductal adenocarcinoma (PDAC) cell interactions with collagens. We found that while well-differentiated cells use *α*2 exclusively to adhere and migrate on collagenI, poorly differentiated PDAC cells demonstrate reduced reliance on, or complete loss of, *α*2. Since well-differentiated PDAC lines exhibit reduced *in vitro* invasion and *α*2-blockade suppressed invasion of well-differentiated lines exclusively, we hypothesized that *α*2 may suppress the malignant phenotype in PDAC. Accordingly, ectopic expression of *α*2 retarded *in vitro* invasion and maintenance on collagenI exacerbated this effect. Affymetrix profiling revealed that kallikrein-related peptidase-5 (KLK5) was specifically upregulated by *α*2, and reduced *α*2 and KLK5 expression was observed in poorly differentiated PDAC cells *in situ*. Accordingly, well-differentiated PDAC lines express KLK5, and KLK5 blockade increased the invasion of KLK5-positive lines. The *α*2-cytoplasmic domain was dispensable for these effects, demonstrating that the *α*2-ectodomain and KLK5 coordinately regulate a less invasive phenotype in PDAC.

## 1. Introduction

Pancreatic ductal adenocarcinoma (PDAC) has one of the highest mortality rates of all cancers; although it accounts for only 2% of new cancer cases each year in the United States, it is the fourth leading cause of cancer mortality [1]. Despite this, the biology of PDAC remains poorly understood. Mutations associated with PDAC initiation have allowed the development of a timeline of PDAC etiology [2]; however, factors contributing to the progression of the disease are less well defined. PDAC is associated with prominent desmoplasia, which is characterized by significant deposition of collagen I, II, and IV [3]. The collagen-binding *α*2-integrin (*α*2) is expressed by both normal pancreatic ductal epithelium and PDAC *in situ* [4, 5], and previous studies have implicated the *α*2*β*1 integrin as the primary collagen receptor in PDAC cells [6]. However, immunohistochemical studies have failed to demonstrate a consistent pattern of *α*2 expression in PDAC *in situ* [3–5, 7, 8], complicating the determination of *α*2's role in PDAC etiology and/or progression. Importantly, while well-differentiated, poorly metastatic PDAC cells demonstrate *α*2-dependent responses to collagenI, poorly differentiated and highly metastatic MIAPaCa2 cells lack collagenI interactions altogether [6]. Moreover, *α*2 is associated with maintenance of tissue architecture and cellular differentiation in other epithelial tissues [9]. Indeed, while well-differentiated *α*2-positive PDAC cells have been shown to produce extensive primary tumors that invade locally, poorly differentiated *α*2-negative PDAC cells produce small primary tumors with prominent distant dissemination [10]. Thus, while *α*2-positive pancreatic epithelial cells utilize this integrin for collagenI interactions, overcoming this interaction may be advantageous to malignant PDAC cells, especially in the context of the collagen-rich desmoplastic reaction that characterizes PDAC *in situ*.

We assessed the role of *α*2 in regulating the invasion of PDAC cells *in vitro*. Our data demonstrate that while *α*2 is a key mediator of invasion in well to moderately differentiated PDAC cells, these cells exhibit lesser invasion that is largely kallikrein-related peptidase (KLK) dependent. In contrast, highly metastatic, poorly differentiated PDAC cells demonstrate higher levels of *in vitro* invasion that are largely *α*2 and KLK independent. We further implicate the *α*2 ectodomain in mediating this phenotype and demonstrate that continued exposure to collagenI exacerbates the *α*2-dependent invasion-suppressor effect, indicating that the presence of *α*2 would be additionally inhibitory to cells continuously exposed to the collagen-rich desmoplasia that is characteristic of PDAC *in situ*.

## 2. Materials and Methods

### 2.1. Cells

CAPAN1, CAPAN2, BxPC3, MIAPaCa2, and Panc1 cells were originally from ATCC, and cultured according to ATCC. Immortalized, untransformed HPDE-E6E7c7 (HPDE) cells were provided by M. Tsao (Toronto Health Network, ON, Canada) and cultured in keratinocyte media supplemented with EGF and pituitary extract (Invitrogen, Carlsbad, CA, USA). PT45P1 cells were a generous gift of H. Kalthoff (Kiel, DE), and cultured in RPMI/10% fetal bovine serum (FBS). COLO357 cells were provided by M. Korc (UCI, Irvine, CA, USA) and cultured in DMEM/10% FBS. Serum-free (SF) medium consisted of all components except serum, as appropriate for the cell line, supplemented with 0.5% bovine serum albumin (BSA). Cell line differentiation characteristics are described in Supplemental Table S1 available online at doi: 10.1155/2011/365651. 

### 2.2. Antibodies and Reagents

Function-blocking anti-integrin antibodies were from Santa Cruz Biotechnology (Santa Cruz, CA, USA) or Chemicon/EMD (San Diego, CA, USA) and include: *α*1(5E8D9); *α*2(P1H5); *α*3(P1B5); *β*1(P4C10). Anti-*α*2 mAb (611016) used for immunoblotting and IHC was from BD Transduction Laboratories (Lexington, KY). Goat anti-hKLK5 (AF1108) and anti-hKLK6 (AF2008) affinity purified pAbs were from R&D Systems (Minneapolis, MN). Anti-actin was from Sigma (St. Louis, MO). Purified bovine collagenI (Purecol) was from Advanced Biomatrix (San Diego, CA, USA). Peptide-based kallikrein inhibitor (RP10161) was from GenScript (Piscataway, NJ, USA). HRP- and FITC-conjugated secondary antibodies were from Jackson Immunochemicals (West Grove, PA, USA). pGEX vector encoding GST-tagged tenascin FN_III_ domain was generously provided by K. Crossin (TSRI, La Jolla, CA, USA), and produced as described previously [11].

### 2.3. Expression Constructs and Transfection/Selection

pBS(KS+) containing wildtype human *α*2-integrin CDS (clone 2.72F) was from ATCC. The *α*2 CDS was excised with KpnI and ligated into pCDNA3.1(zeo) to create pCDNA3.1/*α*2. The *α*2Δcyto insert was created by PCR of pCDNA3.1/*α*2 with T7 sequencing primer (forward) and *α*2Δcyto/XbaI primer (Supplemental Table S2), which contains a 3^'^ stop codon and XbaI site. The resulting product was digested with XbaI and shuttled through pEF4a. The insert was excised from pEF4a with KpnI and NotI and inserted into these sites on pCDNA3.1(zeo). pCDNAIneo/*α*9DM1 and pCDNAIneo/*α*9*α*2 were generously provided by D. Sheppard (UCSF, San Francisco, CA, USA), and have been described previously [11]. KLK5 and KLK6 CDS's were amplified from COLO357 cDNA using primers containing engineered XbaI restriction sites (Supplemental Table S2). The resulting products were cut with XbaI and inserted into the XbaI site of pCDNA3.1(zeo) immediately upstream of an IRES linked to a hygromycin phosphotransferase gene (base construct described previously; [12]). Sequences were verified at the Moores UCSD Cancer Center DNA Sequencing Shared Resource. MIAPaCa2 cells were transfected according to the manufacturer's instructions for Lipofectamine2000 (Invitrogen, Carlsbad, CA, USA). MP2/*α*2 and MP2/*α*2Δcyto cells were panned on plates coated with 25 *μ*g/mL collagenI and blocked with 5% BSA/PBS, and selected with zeocin. MP2/*α*9DM1 and MP2/*α*9*α*2 cells were panned and maintained in the presence of G418 on 10 *μ*g/mL GST-TnFN_III_. KLK-expressing cells were selected with hygromycin. Stable cell populations were maintained in the presence of selection agent. Transient transfections employed cotransfection of pEF4-LacZ and x-gal staining to identify transfected cells.

### 2.4. Immunoblotting

Cells were lysed on the plate in NP40 lysis buffer (50 mM Tris pH 7.4, 150 mM NaCl, 1% NP-40) containing Complete Protease Inhibitor Cocktail supplemented with 10 mM PMSF, 1 mM NaF, and 10 mM Na_3_VO_4_. Samples were prepared and analyzed as described previously [12].

### 2.5. Enzyme-Linked Immunosorbent Assay (ELISA)

Equal volumes of conditioned medium were coated in triplicate onto 96-well microtiter plates. Wells were blocked with 0.5% gelatin prior to incubation with primary antibody. Wells were washed with TBS-T and incubated with HRP-conjugated secondary antibody. Antibody complexes were detected with the peroxidase substrate SureBlue TMB (KPL; Gaithersburg, MD). The reaction was stopped with 0.2 N HCl and absorbance read at 450 nm.

### 2.6. Flow Cytometry

Flow cytometry was performed on a FACScalibur (BD Biosciences, Bedford, MA, USA) at the Moores UCSD Cancer Center Flow Cytometry Shared Resource. Gates were set with cells treated with FITC-conjugated secondary antibody alone, and 5 *μ*g/mL propidium iodide was included to exclude dead and dying cells.

### 2.7. Adhesion Assay

Subconfluent cells were seeded in SF-media at 2.5 × 10^5^ cell per 48-well nontissue culture-treated plate that had been coated with 25 *μ*g/mL (unless indicated otherwise) collagenI and blocked with 5% BSA/PBS. Cells were allowed to adhere for 45 min (unless otherwise indicated) before washing, staining with 1% Toluidine Blue and manual enumeration or extraction of dye with 10% acetic acid and quantitation of absorbance at 595 nm. Where indicated, cells were preincubated with antibodies (50 *μ*g/mL) for 5 min before seeding into wells.

### 2.8. Migration Assay

Millicell sterile culture plate inserts (8 *μ*m pore; Millipore Corp., Billerica, MA, USA) were coated on the underside with 20 *μ*g/mL collagenI prior to insertion into culture plates containing SF media. Cells (2.5 × 10^5^) were added in SF-media to the upper chamber in the presence or absence of 50 *μ*g/mL function-blocking antibodies at 37°C. Inserts were fixed at the indicated times, stained with 1% Toluidine Blue, and unmigrated cells removed and migrated cells enumerated. Where indicated, antibodies (50 *μ*g/mL) were present in both chambers.

### 2.9. Invasion Assay

Subconfluent cells (2.5 × 10^5^) were seeded in SF-media into BioCoat Growth Factor-Reduced Matrigel Invasion Chambers (BD Biosciences, Bedford, MA, USA) in wells containing full growth media. Chambers were incubated at 37°C for 24 h (48 h for COLO357 cells), or as indicated before fixing, staining with 1% Toluidine Blue, removal of uninvaded cells and manual enumeration. Where indicated, antibodies (50 *μ*g/mL) were present in both chambers.

### 2.10. Reverse Transcription-PCR

cDNA was synthesized from 1 *μ*g of total RNA using oligo-dT primer. PCR was performed on 1 *μ*L of total cDNA using primers described in Supplemental Table S2.

### 2.11. Immunohistochemistry

Tissue samples were obtained under approved Institutional Review Board protocol from the UCSD Dept. of Pathology archives. Samples were deparaffinized, rehydrated, and incubated with 1% H_2_O_2_ to inactivate endogenous peroxidases. Slides were quenched with 50 mM glycine, blocked with 2% horse serum/5% BSA/phosphate-buffered saline (PBS), pH 7.4, and renatured using Target Retrieval Solution (DAKO North America; Carpinteria, CA, USA) prior to incubation with anti-*α*2 or anti-hKLK5 pAb at 2.5 *μ*g/mL. Slides were washed and biotinylated antimouse or antigoat applied according to the VectaStain Elite ABC Kit (Vector Labs; Burlingame, CA, USA). Sections were developed with DAB, counterstained with hematoxylin, dehydrated and mounted.

### 2.12. Affymetrix Gene Array Analysis

Detailed in the Supplementary Material section along with tables of representative gene changes (Supplemental Tables S3 and S4). The full set of array data is available in the GEO repository via Accession no. GSE18277 (http://www.ncbi.nlm.nih.gov/geo/query/acc.cgi?token=zreftooauqiuupq&acc=GSE18277).

### 2.13. Statistics

Gene array statistics are described in Supplemental Methods. Adhesion, migration and invasion were analyzed by two-tailed Students *t*-test.

## 3. Results

### 3.1. Pancreatic Collagen-Binding Integrin Expression Reflects Differentiation State

**Figure 1:**
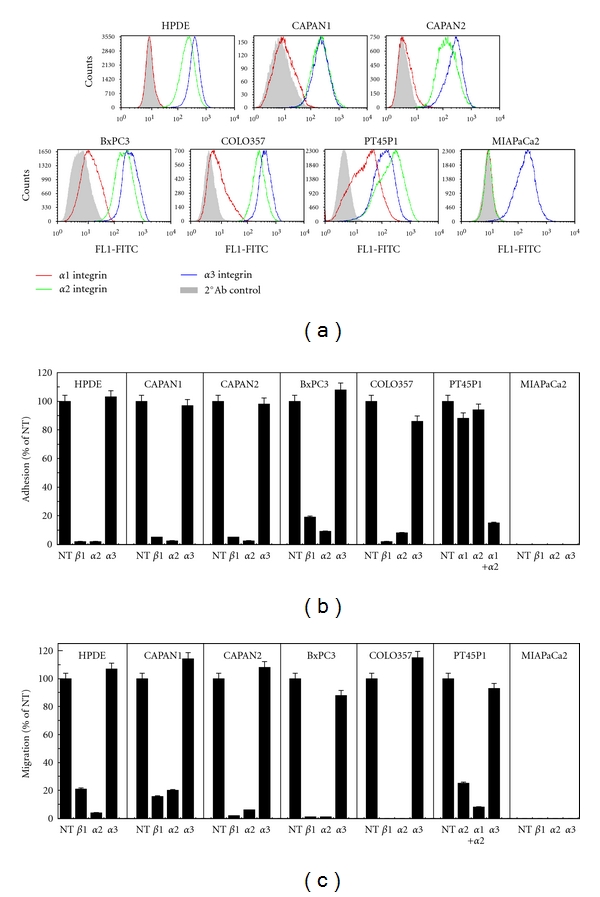
PDAC integrin expression and utilization reflects cellular differentiation. (a) Flow cytometric analysis of *α*1, *α*2, or *α*3 surface expression in a spectrum of pancreatic ductal cells including untransformed (HPDE), well-(CAPAN1 and CAPAN2), moderately (BxPC3, COLO357) and poorly differentiated PDAC (PT45P1 and MIAPaCa2) (see Supplemental Table S1 for cell characteristics). Secondary antibody controls, solid. (b) Adhesion of the cells in (a) to 25 *μ*g/mL collagenI for 45 minutes in the presence or absence of function-blocking antibodies to the indicated integrins, as described in Materials and Methods. (c) Serum-free migration (16 h) through Transwell inserts coated on the underside with 20 *μ*g/mL collagenI in the presence or absence of function-blocking antibodies as in (b).

Flow cytometric analysis of pancreatic cells that span the spectrum of differentiation (see Supplemental Table S1) demonstrated a distinct pattern of collagen-interactive integrin surface expression *in vitro* (Figure 1(a)). All cells express significant *α*3 integrin. However, although untransformed HPDE and well-differentiated CAPAN1 (G1) and CAPAN2 (G1) cells demonstrated significant *α*2 expression and no *α*1 expression, moderately differentiated BxPC3 (G2) and COLO357 (G2) cells express significant *α*2 and marginal *α*1. In contrast, poorly differentiated PT45P1 (G3/G3+) cells express significant levels of both *α*1 and *α*2, while very poorly differentiated MIAPaCa2 (G3+) cells lack both integrins altogether. The collagen-binding integrins *α*10 and *α*11 were not studied due to their restricted expression and the fact that all collagenI interactions in our cells could be accounted for by *α*1 and *α*2. These data demonstrate the differential expression of collagen-interactive integrins in a manner coincident with differentiation.

### 3.2. Poorly Differentiated PDAC Cells Demonstrate Reduced Reliance or Complete Loss of *α*2 Integrin Interactions

Untransformed HPDE cells as well as well- and moderately differentiated PDAC cells demonstrated complete reliance on the *α*2*β*1 integrin for adhesion and migration on collagenI (Figures 1(b) and 1(c)). In contrast, *α*2-blockade of poorly differentiated PT45P1 cells had little effect on their adhesion to collagenI. Indeed, while *α*1- (Figure 1(b)) and *α*3-blockade (not shown) similarly failed to suppress binding, combination of *α*1- and *α*2-function-blocking antibodies almost completely abrogated PT45P1 adhesion to collagenI (Figure 1(b)). Although *α*2-blockade significantly suppressed PT45P1 migration on collagenI, combined blockade with *α*1 promoted enhanced suppression (Figure 1(c)), further demonstrating the reduced reliance of these poorly differentiated cells on *α*2 for collagen interactions. Importantly, very poorly differentiated MIAPaCa2 cells demonstrate no adhesion or migration on collagenI (Figures 1(b) and 1(c)), commensurate with their lack of *α*1 and *α*2 integrin expression and similar to previous reports [6].

### 3.3. Ectopic Expression of *α*2 in MIAPaCa2 Cells Recapitulates Collagen Interactions

**Figure 2:**
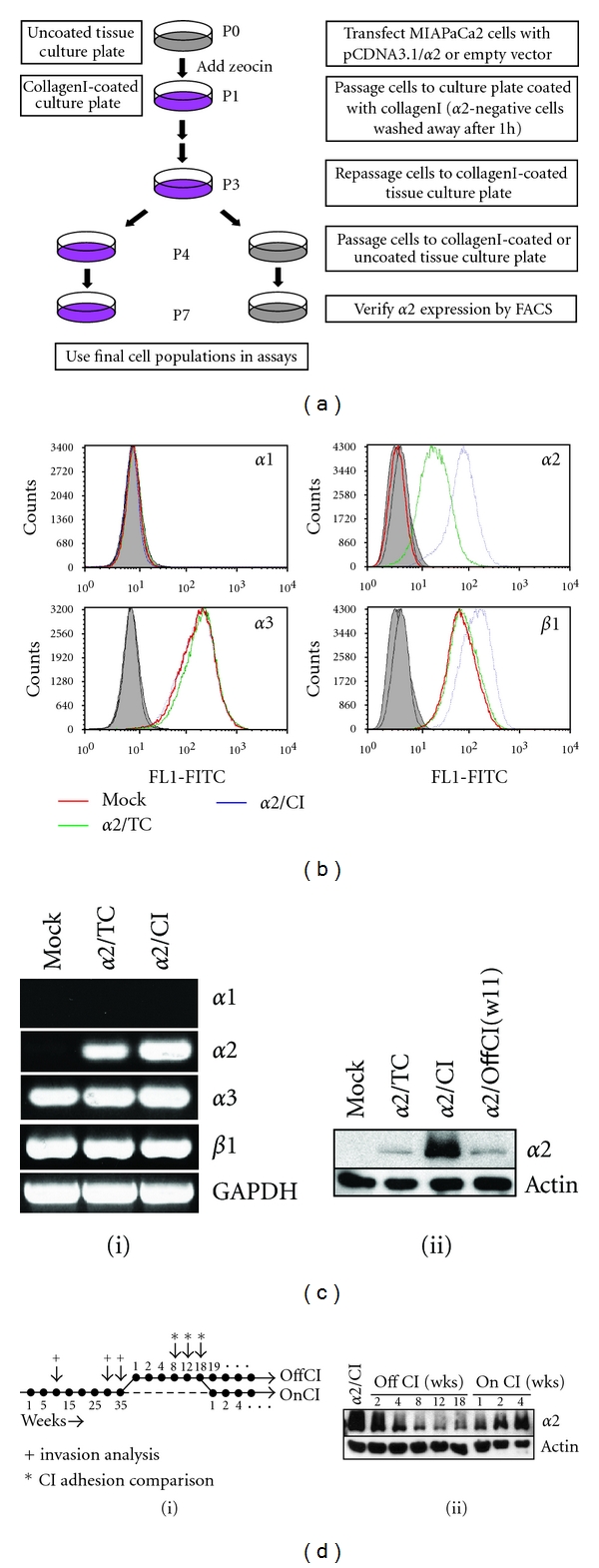
Reexpression of *α*2 in MIAPaCa2 Cells. (a) Expression and selection scheme. (b) Flow cytometric analysis of cell surface *α*1, *α*2, *α*3 and *β*1 integrin expression in MP2-mock, MP2-*α*2/TC, and MP2-*α*2/CI cells. Secondary antibody controls (solid) are overlapped. (c) Integrin levels were assessed at the transcript (i) and total protein level (ii) using RT-PCR and immunoblotting as described in Materials and Methods. GAPDH and actin, controls. (d) The effect of maintenance on collagenI was assessed by replating cells grown on collagenI for an extended period into standard tissue culture conditions and then returning those cells to collagenI culture at the indicated times (i). (ii) Lysates were immunoblotted for total *α*2 expression. Actin, control.

Since MIAPaCa2 cells lack *α*2 integrin and do not adhere to collagenI, we ectopically expressed *α*2 under a viral (cytomegalovirus) promoter and maintained one population on collagenI (MP2-*α*2/CI), and one population in standard tissue culture (MP2-*α*2/TC) (Figure 2(a)). MP2-*α*2/CI cells demonstrate significantly higher *α*2 integrin surface levels than MP2-*α*2/TC cells (mean FL1 77.0 versus 31.3) (Figure 2(b)), and this higher surface expression is reflected in transcript as well (Figure 2(c)(i)). Similarly, whereas mock and *α*2 transfectants demonstrate identical *β*1 levels in culture, repeated passage on collagenI promotes significantly higher levels of surface *β*1 subunit (mean FL1 136.7 versus 79.3) (Figure 2(b)), likely due to the requirement for heterodimerization to facilitate translocation of the *α*2 subunit to the cell surface. All cells express identical *α*3 levels and lack *α*1 expression (Figures 2(b) and 2(c)(i)), indistinguishable from the parental cells (Figure 1(a)). Since integrins are often retained in an intracellular pool, the differential surface expression observed may not reflect different total levels of protein, but rather the preferential export of *α*2*β*1 to the cell surface in response to the demands of continued growth on a collagen substratum. However, immunoblotting of whole cell lysates demonstrated significantly more *α*2 in the MP2-*α*2/CI cells than MP2-*α*2/TC cells (Figure 2(c)(ii)). This differential expression was not the result of selecting a high-expressing population during initial panning, since both populations were derived from the same original panning process (Figure 2(a)). Moreover, the active regulation of *α*2 levels by exposure to collagenI is further demonstrated by the reduction in *α*2 expression noted upon removal of MP2-*α*2/CI from collagenI and culture under standard conditions as well as the heightened expression that results from repassaging the cells onto a collagenI-coated substratum (Figure 2(d)(i, ii)).

### 3.4. Differential *α*2 Expression Promotes Dose-Dependent CollagenI Interaction and Suppression of Invasion

**Figure 3:**
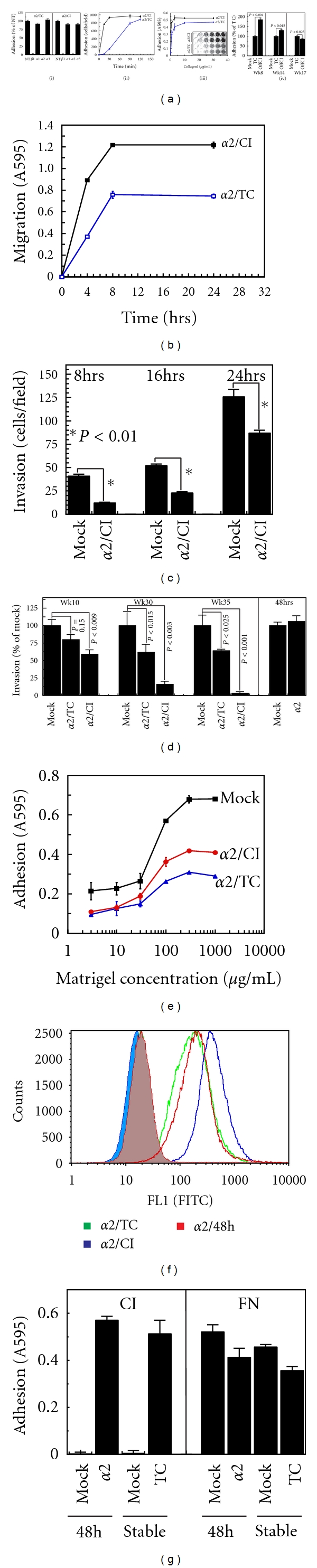
Reexpression of *α*2 recapitulates collagen interactions and retards invasion. (a) (i) Adhesion of MP2-*α*2/TC and MP2-*α*2/CI cells in the presence or absence of function-blocking antibodies directed towards the indicated integrins. (ii) Time course of adhesion of MP2-*α*2/TC and MP2-*α*2/CI cells. (iii) Titration of collagenI adhesion of MP2-*α*2/TC and MP2-*α*2/CI cells. (iv) Adhesion of MP2-mock and MP2-*α*2/TC cells to collagenI was compared to the collagenI adhesion of MP2-*α*2/CI cells that had been grown for the indicated times off collagenI (OffCI). (b) Haptotactic migration time course of MP2-*α*2/TC and MP2-*α*2/CI cells on collagenI. (c) *In vitro* invasion of MP2-mock and week10 MP2-*α*2/CI cells. (d, left panel) Invasion of MP2-mock and MP2-*α*2/TC cells was compared with MP2-*α*2/CI cells after the indicated weeks of culture. (d, right panel) Parental MIAPaCa2 cells were cotransfected pEF4/LacZ reporter and either empty pCDNA3.1 vector (Mock) or the pCDNA3.1/*α*2 construct, and invasion of LacZ-positive cells examined 48 hours later. (e) Adhesion of MP2-mock, MP2-*α*2/TC, and MP2-*α*2/CI cells to a titration of Matrigel. (f) FACS analysis of *α*2 expression by transiently transfected MP2 cells (*α*2/48 h) versus stable MP2-*α*2/TC and MP2-*α*2/CI cells. (g) CollagenI (CI) and fibronectin (FN) adhesion of mock- or *α*2-transfected MP2 cells at 48 h versus stable MP2-mock and MP2-*α*2/TC cells.

Both MP2-*α*2/TC and MP2-*α*2/CI cells demonstrate complete reliance on integrin *α*2*β*1 for adhesion to collagenI (Figure 3(a)(i)). However, the higher *α*2 expression of MP2-*α*2/CI cells translates to more rapid and more complete collagenI adhesion (Figure 3(a)(ii)), and a requirement for less collagenI to achieve maximal adhesion (Figure 3(a)(iii)). Moreover, MP2-*α*2/CI cells transitioned to standard tissue culture conditions demonstrated a progressive loss of this enhanced adhesiveness to collagenI (Figure 3(a)(iv)). The elevated *α*2 expression and collagenI adhesion of the MP2-*α*2/CI cells also translated to a higher rate and overall level of haptotactic migration on collagenI (Figure 3(b)); however, MP2-*α*2/CI cells actually demonstrated a reduced rate of invasion compared to MP2-*α*2/Mock (Figures 3(c) and 3(d)) and MP2-*α*2/TC (Figure 3(d)). Importantly, MP2-*α*2/TC and MP2-*α*2/CI cells demonstrated a time-dependent effect whereby more invasion suppression was achieved after prolonged culture, culminating in a maximum of 40% reduction for MP2-*α*2/TC cells and >90% for MP2-*α*2/CI cells versus MP2-*α*2/Mock cells (Figure 3(d)). Although these data and those shown in Figures 2(c) and 2(d) could be interpreted to mean that higher levels of *α*2 merely translate to enhanced adhesion to Matrigel, thus resulting in decreased invasion, the opposite is actually true, as MP2-*α*2/TC and MP2-*α*2/CI adhesion to Matrigel is actually retarded versus MP2-*α*2/mock cells (Figure 3(e)). As might be predicted from the time-dependent nature of the anti-invasive effect, transient expression of *α*2 did not retard the invasion of parental MIAPaCa2 cells (Figure 3(d), right panel), although these cells expressed similar levels of surface *α*2 as MP2-*α*2/TC and MP2-*α*2/CI cells (Figure 3(f)) and exhibited significant *de novo* adhesion to collagenI (Figure 3(g)).

### 3.5. *α*2 Regulates the Expression of Invasion-Related Gene Products That Retard Invasion *In Vitro*

**Figure 4:**
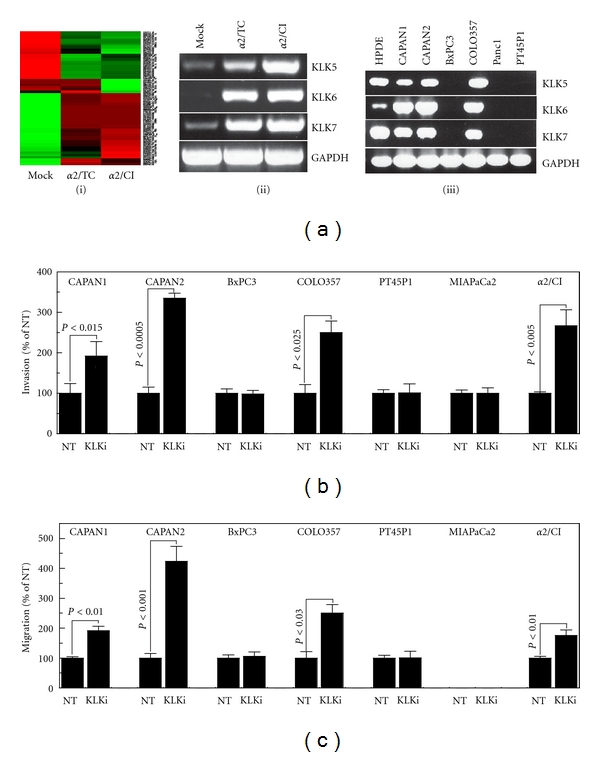
Stable *α*2 reexpression upregulates KLK-5, 6, and 7, which are expressed in a differentiation-dependent manner and regulate *in vitro* invasion and collagenI migration. (a) (i) Heatmap presentation of identified gene products. (ii) RT-PCR analysis of Affymetrix-identified candidates KLK-5, 6, and 7 in MP2-mock, MP2-*α*2/TC and MP2-*α*2/CI cells. GAPDH, control. (iii) RT-PCR analysis of KLK-5, 6 and 7 expression in a spectrum of pancreatic cells including: untransformed (HPDE), well-(CAPAN1 and CAPAN2), moderately (BxPC3 and COLO357) and poorly differentiated PDAC (Panc1 and PT45P1) (see Supplemental Table S1 for information on cellular grade and origin). GAPDH, control. (b) Invasion analysis of the indicated cells in the presence or absence of KLK inhibitor (KLKi). (c) CollagenI migration analysis of the indicated cells in the presence or absence of KLK inhibitor (KLKi).

The data shown in Figure 3(d) demonstrate the requirement for long-term expression of the *α*2 integrin to achieve maximal invasion-suppressive effects, and the fact that transient expression of *α*2 had no effect on invasion. Since this effect is not due to increased Matrigel adhesion (Figure 3(e)), this suggests the need for gene expression changes to effect the invasion suppression phenotype of *α*2. Therefore, we performed Affymetrix global gene expression analysis to determine transcripts regulated by *α*2 expression (*α*2/TC), or *α*2 expression coupled with constitutive engagement (*α*2/CI). Over 6,000 transcripts were differentially expressed at a false discovery rate (FDR) ≤0.01 (Figure 4(a)(i), Supplemental Table S3). Among the most prominently affected were kallikrein-related peptidases (KLKs) 5, 6 and 7, which have a relatively ill-defined relationship to the regulation of cell migration and invasion. We verified the upregulation of these KLK transcripts in MP2-*α*2/TC cells, and the exacerbated expression in MP2-*α*2/CI cells (Figure 4(a)(ii)). We further verified the expression of KLK-5, 6 and 7 transcripts by untransformed and well-to moderately differentiated PDAC cell lines, to the exclusion of poorly differentiated PDAC lines (Figure 4(a)(iii)). The sole exception to this pattern was moderately differentiated (G2) BxPC3 cells, which did not express detectable KLK-5, 6 or 7 transcript. In all cases, KLK-5, 6, and 7 were coordinately expressed; that is, either all three were expressed or none were. Affymetrix demonstrated that no other KLKs were expressed by these cells. Importantly, blockade of KLK activity with a peptide-based inhibitor enhanced the invasion of endogenously KLK-positive cells as well as the MP2-*α*2/CI cells, to the exclusion of KLK-negative cells (Figure 4(b)), and KLK blockade had a similar effect on haptotactic migration of KLK-positive cells towards collagenI (Figure 4(c)).

The enzymatic specificities of KLK5 and KLK6 differ dramatically from KLK7. The major determinant of specificity for KLK-5, 6, and 7 is the S1 pocket, demonstrating primary specificity definition at the P1 position, with additional specificity preference dictated by the P2 position; for different reasons, the P3 and P4 positions are relatively unimportant in defining the specificities of KLK-5, 6 and 7 [13]. As such, KLK5 and KLK6 exhibit trypsin-like specificity with a strong preference for Arg at the P1 position of substrates (KLK5, R ≫ K, but not Y or F; KLK6, R ≫ A, M > K), while KLK7 exhibits a unique chymotrypsin-like specificity for Tyr at P1 (Y > A, M *⋙* F, R, K), and also at P2 (Y > L, T, M, F) [13]. Therefore, it is highly unlikely that the peptide-based inhibitor used in Figure 4 (PFR∣SVQ) would affect KLK7, but the Arg at the P1 position of this peptide would serve as relatively optimal for binding to the substrate-binding S1 pockets of KLK5 and KLK6.

**Figure 5:**
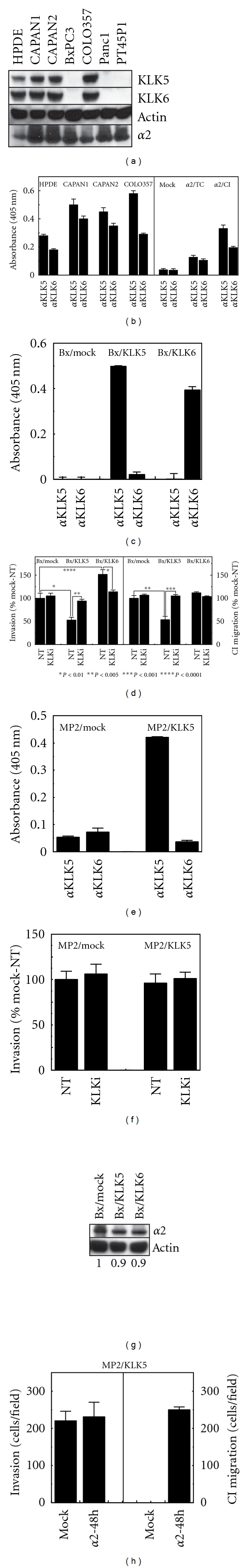
KLK5 mediates an anti-invasion phenotype in *α*2-expressing PDAC cells *in vitro*. (a) Immunoblot analysis of the indicated cells for expression of KLK5, KLK6, and *α*2 integrin. Actin, loading control. (b) ELISA of KLK5 and KLK6 secretion into culture media conditioned by the indicated cells. (c) ELISA analysis of KLK5 and KLK6 secretion by engineered BxPC3 cells stably transfected with KLK5 (Bx/KLK5) and KLK6 (Bx/KLK6) versus hygromycin-resistant mock transfectants (Bx/mock). (d) Invasion (left panel) and collagenI migration (right panel) analysis of the indicated stable BxPC3 populations in the presence or absence of KLK inhibitor (KLKi). (e) ELISA analysis of KLK5 secretion by engineered MP2 cells stably transfected with KLK5 (MP2/KLK5) versus hygromycin-resistant mock transfectants (MP2/mock). *α*KLK6, control. (f) Invasion analysis of the indicated stable MP2 cells in the presence or absence of KLK inhibitor (KLKi). (g) Immunoblot analysis of total *α*2 expression by the indicated stable BxPC3 populations. Actin, control. (h) Invasion (left panel) and collagenI migration (right panel) analysis of the stable MP2/KLK5 cells transiently transfected with *α*2 (*α*2-48 h) or empty vector (mock).

Based on this information, we further assessed the production of KLK5 and KLK6 by the spectrum of cells that we had verified at the transcript level. Consistent with their RNA profile, untransformed HPDE and all well- to moderately differentiated PDAC except BxPC3 expressed significant quantities of both KLK5 and KLK6 (Figure 5(a)). Total cellular *α*2 expression is similar between the cell lines, similar to the surface expression shown in Figure 1(a). Again, poorly differentiated cells proved negative for both KLK5 and KLK6. We further verified the secretion of KLK5 and KLK6 in the cells that showed protein at the whole cell lysate level (Figure 5(b), left panel), as well as in the MP2-*α*2/TC and MP2-*α*2/CI cells, to the exclusion of the MP2-mock cells (Figure 5(b), right panel). These data demonstrate that KLK5/6 expression correlates well with grade, is driven by stable *α*2 reexpression in MP2 cells, and further indicate the availability of *α*2-positive cells suitable for engineering ectopic KLK expression (i.e., BxPC3). 

To assess the KLK involved in regulating the invasive phenotype of these cells, we stably transfected BxPC3 cells with hKLK5 (Bx/KLK5) or hKLK6 (Bx/KLK6) under the control of a CMV promoter and linked via an IRES to a hygromycin phosphotransferase gene, which links hygro-resistance to KLK expression. Stable heterogenous populations were selected and found to secrete similar quantities of KLK5 and KLK6 as endogenously KLK5/6-positive cells (Figure 5(c)). Mock-transfected (Bx/mock) cells resistant to hygromycin did not secrete detectable KLK5 or KLK6. We further found that Bx/KLK5 cells demonstrated reduced *in vitro* invasion (Figure 5(d), left panel) and haptotactic migration towards collagenI (Figure 5(d), right panel) compared to Bx/mock cells. Consistent with the findings of a prior report [14], Bx/KLK6 cells actually invaded better than Bx/mock cells. Importantly, the KLK inhibitor reversed both phenotypes, demonstrating the specificity of the effect and the efficacy of the inhibitor against both KLK5 and KLK6 and further suggesting that the net effects of KLK5 outweigh those of KLK6 in this cell system. Having identified KLK5 as the KLK responsible for at least part of the observed anti-invasive phenotype, we stably transfected the *α*2-negative parental MP2 cells with hKLK5 (MP2/KLK5 cells) or empty vector (MP2/mock cells). Stable heterogeous populations were selected and MP2/KLK5 cells were found to secrete quantities of KLK5 similar to endogenously positive as well as stable Bx/KLK5 cells (Figure 5(e)). MP2/mock cells did not secrete KLK5 and neither population secreted KLK6. Surprisingly, MP2/KLK5 cells did not demonstrate reduced *in vitro* invasion versus MP2/mock cells, nor was their invasion affected by the KLK inhibitor (Figure 5(f)). Based on these data, we questioned whether *α*2 expression was necessary for the KLK5-dependent anti-invasive phenotype. It should be noted that stable expression of KLK5 and KLK6 did not significantly affect the expression of *α*2 in BxPC3 (Figure 5(g)) or MP2 cells, which remained *α*2-negative (not shown). To determine if transient expression of *α*2 could rescue the KLK5 phenotype, we transfected stable MP2/KLK5 cells with the *α*2 expression construct or empty vector. After 48 h the *α*2-transfected MP2/KLK5 cells demonstrated identical invasion to mock-transfectants (Figure 5(h), left panel); however, the transfected cells demonstrated significant *de novo* migration towards collagen I (Figure 5(h), right panel). These data suggest that long-term expression of *α*2 is likely required for the full phenotype, either directly or indirectly. We did not assess stable expression of *α*2 in the MP2/KLK5 cells, since stable *α*2 expression would lead to the upregulation of KLK-5, 6, and 7, as shown previously (Figures 4(a)(ii) and 5(b)). 

**Figure 6:**
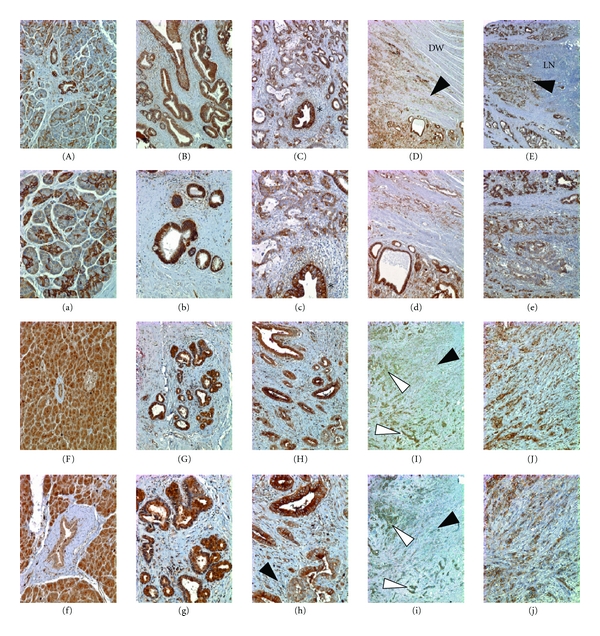
*α*2 and KLK5 are expressed in normal pancreatic ductal epithelium and reduced or absent in poorly differentiated PDAC. (Upper Panels) (A) Normal pancreas stained for *α*2 showing positive ducts and ductules. (a) Higher power image of (A). (B) Well-differerentiated PDAC showing *α*2 positivity. (b) Higher power image of (B). (C) Moderate- to poorly differentiated PDAC showing reduction/loss of *α*2 in the more poorly differentiated cells. Asterisk denotes a benign reactive duct within the tumor region. (c) Higher power image of (C). (D) Poorly differentiated *α*2-reduced/negative PDAC cells (black arrowhead) invading the wall of the duodenum (DW) with adjacent *α*2-positive noninvading well-differentiated ducts. (d) Higher power image of (D). (E) Poorly differentiated *α*2-reduced/negative PDAC cells (black arrowhead) invading the lymph node (LN), with adjacent *α*2-positive non invading well-differentiated ducts. (e) Higher power image of (E). (Lower panels) (F) Normal pancreas stained for KLK5 showing ducts and ductules positive (in addition to acinar cells and some signal in islets). (f) Higher power image of (F). (G) Well-differentiated ducts showing KLK5 positivity. (g) Higher power image of (G). (H) Well- to moderately differentiated KLK5-positive ducts with adjacent less differentiated, KLK5-reduced/negative cells (black arrowhead). (h) Higher power image of (H). (I) A field of poorly differentiated/anaplastic PDAC cells (black arrowhead) with adjacent clusters of KLK5-positive cells (white arrowheads); low power. (i) *α*2 staining of a serial section of (I). (J) Higher power image of KLK5 staining in I. (j) Higher power image of *α*2 staining in (i).

Previous immunohistochemical studies have failed to demonstrate a consistent pattern of *α*2 expression in PDAC *in situ* [3–5, 7, 8]. To assess the relevance of our findings with regard to the human condition, we assessed the expression of *α*2 and KLK5 in patient samples spanning the spectrum of normal to well-, moderately, and poorly differentiated PDAC. Consistent with prior reports, we found that *α*2 is strongly and specifically expressed in ductal epithelial cells of the normal pancreas, including large and small ducts and the ductules servicing acinar clusters (Figures 6(A) and 6(a)). Expression of *α*2 is uniformly maintained in well differentiated PDAC lesions (Figures 6(B) and 6(b)) but progressively lost in more poorly differentiated cells, even those adjacent to better differentiated PDAC cells (Figures 6(C) and 6(c)). Importantly, *α*2-reduced/negative cells were observed invading structures including the wall of the duodenum (Figures 6(D) and 6(d)) and regional lymph nodes (Figures 6(E) and 6(e)), adjacent to *α*2-positive well-to moderately differentiated PDAC that failed to invade these structures. Indeed, while we observed no heterogeneity of *α*2 staining in normal (*n* = 8) and well-/moderately differentiated samples (*n* = 16), we observed reduced staining of *α*2 in 62.5% of poorly differentiated samples (*n* = 8). Consistent with a prior report [15], we found strong expression of KLK5 in the acinar cells of the normal pancreas, but also in the large and small ducts as well as the ductules servicing acinar clusters (Figures 6(F) and 6(f)). Similar to our observations with *α*2, KLK5 expression was uniformly maintained in well-differentiated PDAC lesions (Figures 6(G) and 6(g)), but progressively lost in more poorly differentiated cells, even those adjacent to better differentiated PDAC cells (Figures 6(H) and 6(h)). Importantly, staining of serial sections demonstrated that KLK5-positive (Figure 6(I, J)) clusters of interactive cells were also *α*2-positive (Figures 6(i) and 6(j)), while poorly differentiated/anaplastic PDAC cells invading into the stroma demonstrated significantly reduced expression or loss of KLK5 (Figures 6(I) and 6(J)) and *α*2 (Figure 6(i) and 6(j)).

**Figure 7:**
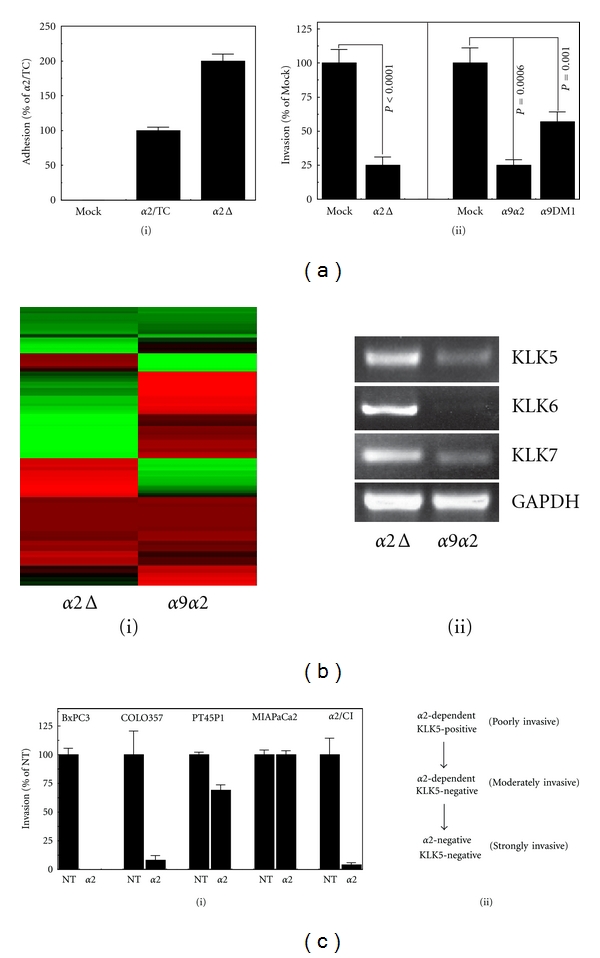
The *α*2 ectodomain regulates expression of KLK-5, 6, and 7, the combination of which mediates an anti-invasion phenotype *in vitro*. (a) (i) CollagenI adhesion of MP2-mock and MP2-*α*2/TC cells compared with MP2-*α*2Δ cells. (ii) Invasion analysis of MP2-mock versus MP2-*α*2Δ cells (left panel), or MP2-mock versus MP2-*α*9*α*2 and MP2-*α*9DM1 cells (right panel). (b) (i) Heatmap presentation of the probeset shown in Figure 4(a)(i) in MP2-*α*2Δ and MP2-*α*9*α*2 cells. (ii) RT-PCR verification of KLK-5, 6 and 7 expression in MP2-*α*2Δ and MP2-*α*9*α*2 cells. (c) (i) Invasion of the indicated cells in the present or absence of function-blocking anti-*α*2 antibody. (ii) Model of PDAC invasion status based on *α*2-dependence and KLK5 expression status.

From a mechanistic standpoint, we questioned which region of *α*2 is responsible for the observed gene regulation and anti-invasive effects. To address this question, we generated a cytoplasmic deletion mutant (*α*2Δ) and stably expressed it in MIAPaCa2 cells (MP2-*α*2Δ). These cells were maintained under drug selection on collagenI. Similar to MP2-*α*2/CI cells, prolonged growth of MP2-*α*2Δ cells on collagenI yielded a collagenI-adhesive phenotype superior to MP2-*α*2/TC cells (Figure 7(a)(i)). To complement the *α*2Δ construct, we stably expressed a *α*9*α*2 chimera that encodes the *α*9 extracellular and transmembrane domains linked to the cytoplasmic domain of the *α*2 integrin in MIAPaCa2 cells (MP2-*α*9*α*2). To control for potential effects of the *α*9 ectodomain, we also expressed a cytoplasmic-deleted *α*9 (MP2-*α*9DMI). These cells were panned and maintained under drug selection on a recombinant FNIII domain of tenascin (TnFN_III_), a specific substrate of the *α*9 integrin [11]. Parental MIAPaCa2 cells lack endogenous *α*9 integrin expression, and do not adhere to TnFN_III_ (not shown). MP2-*α*2Δ cells demonstrated >75% invasion suppression, versus MP2/Mock cells (Figure 7(a)(ii)), and while MP2-*α*9*α*2 cells demonstrated similar invasion suppression, part of this phenotype is likely due to the extracellular domain of the *α*9 integrin, which itself promoted ~43% invasion suppression (MP2-*α*9DM1). Thus, the *α*2 cytoplasmic domain may only be responsible for 32% invasion suppression in these cells. Affymetrix analysis of the MP2-*α*2Δ and MP2-*α*9*α*2 cells demonstrated that many of the gene products identified in Figure 4(a)(i) and Supplemental Table S3 were differentially regulated by either the *α*2 ectodomain (*α*2Δ) or cytoplasmic domain (*α*9*α*2); however, some products were not affected by expression of either construct or were affected by both (Figure 7(b)(i), Supplemental Table S4). Importantly, KLK-5, 6, and 7 were upregulated in MP2-*α*2Δ cells in comparison to MP2-*α*9*α*2 cells (Figure 7(b)(ii)). 

These data demonstrate the regulation of KLK expression by the *α*2 integrin, and the manifestation of a reduced invasion phenotype by the coordinated input of both players. Accordingly, very poorly invasive COLO357 cells that demonstrated KLK-positivity (Figures 4(a)(iii), 5(a), and 5(b)) and a negative effect of KLK's on invasion (Figure 4(b)) also demonstrate complete reliance on *α*2 integrin for invasion *in vitro* (Figure 7(c)(i)). BxPC3 cells also demonstrate complete inhibition of invasion by *α*2-blockade (Figure 7(c)(i)); however, they lack KLK-5, 6, and 7 expression (Figures 4(a)(iii), 5(a), and 5(b)) and are not affected by KLK blockade (Figure 4(b)), which manifests as moderate *in vitro* invasion. In contrast, highly invasive PT45P1 and MIAPaCa2 cells demonstrate loss of reliance on *α*2, or complete lack of effect of *α*2-blockade on invasion, respectively (Figure 7(c)(i)). Importantly, however, the reduced invasion of MP2-*α*2/CI cells is completely suppressed by *α*2-blockade. The specific role of KLK5 in regulating these phenotypes has been demonstrated in Figure 5. These parameters have been summarized in the model shown in Figure 7(c)(ii).

## 4. Discussion

In this study, we demonstrate the regulation of KLK5 expression by the *α*2 integrin and the manifestation of a reduced invasion phenotype by the coordinated input of both players. As such, we propose that the invasion of PDAC cells is at least partly a balance between their reliance on *α*2 for collagen interactions, and their expression and utilization of KLK5, as summarized in the model shown in Figure 7(c)(ii). An invasion-suppressor role for *α*2 is supported by several lines of evidence that implicate *α*2 in the regulation of cellular homeostasis and differentiation. Indeed, the *α*2 integrin has been linked to the maintenance of tissue architecture [16] and the orderly proliferation of normal mammary epithelial cells [9], and a generalized loss of expression has been noted during breast tumor progression *in situ* [17, 18]. More importantly, reexpression of *α*2 in an endogenously *α*2-negative poorly differentiated breast adenocarcinoma line promoted a differentiated epithelial morphology with concomitantly enhanced branching morphogenesis and restoration of contact inhibition of growth *in vitro* and reduced tumor growth *in vivo* [16]. Reciprocal suppression of *α*2 expression in endogenously *α*2-positive well-differentiated breast carcinoma cells retarded branching morphogenesis in a level of expression-dependent manner [16]. Branching morphogenesis has since been shown to be *α*2-dependent using an immortalized, nonmalignant breast epithelial line [16]. These data suggest that *α*2 may actually function as a type of tumor suppressor in the ductal epithelium of the breast.

Importantly, high *α*2 expression has been associated with the orderly proliferation of normal epithelial cells of several tissues [9], and loss of *α*2 retards cyst formation and branching morphogenesis of MDCK epithelial cells [19], consistent with a role in regulating the differentiated epithelial state. Reduced *α*2*β*1 expression is also significantly associated with Duke's stage in colon carcinoma [20]. Indeed, on the basis of a review of the literature, decreased *α*2 expression has been proposed as the single most common change in integrin expression in epithelial malignancies [16]. On the other hand, *α*2 expression was linked to poor survival in patients with advanced melanoma [21], and *α*2*β*1 signaling promotes growth of human prostate cancer cells within bone [22], highlighting the complex nature of *α*2*β*1's role in tumor biology. Indeed, high *α*2 expression promoted collagenI adhesion and migration, as well as invasion of osteosarcoma cells; however, ectopic expression of *α*2 did not support growth of these cells in animals [23]. In PDAC, *α*2*β*1 expression has been observed in normal and well-differentiated tumors, and although a generalized loss of *α*2*β*1 expression has not been reported in more progressed lesions *in situ,* decreased expression and/or changes in subcellular localization have been described [3–5, 7, 8]. Herein, we demonstrate that reduced expression and/or absence of *α*2 by poorly differentiated PDAC cells is relatively common *in situ* and that this often occurs in the context of adjacent strongly *α*2-positive more differentiated PDAC cells. More importantly, our *in vitro* findings provide a potential explanation for the fact that such a generalized loss of expression has not been reported more consistently. In more progressed lesions, the presence of *α*2 integrin may not be indicative of its utilization, as noted for the poorly differentiated PT45P1 cells, which maintain strong *α*2 expression, yet are not dependent upon it for collagen interactions or invasion.

Although *α*2 has been implicated as a potential tumor suppressor in the breast, it has not been specifically shown to regulate invasion *per se*. In our system, reexpression of the *α*2 integrin promotes interaction with collagens, however such reexpression actually reduced cellular invasion *in vitro*. This phenotype, and the accompanying gene expression changes that result from stable *α*2 expression, is exacerbated by maintenance of the cells on collagen in culture, a surrogate for exposure to the significant desmoplasia characteristic of PDAC *in situ*. It should be noted that although maintenance on collagenI has been shown to upregulate total *α*2 levels in endogenously *α*2-positive PDAC cells previously [24], the dynamic regulation of *α*2 expression observed in stable MP2-*α*2 transfectants in response to culture on collagenI is not likely the result of transcription from the endogenous promoter, as the Affymetrix analysis failed to identify *α*2 expression in any of the cells (the transgene construct lacks the 3^'^-UTR sequences used as probes in the analysis). Thus, protein turnover or similar mechanism may be responsible for the altered *α*2 levels observed. More importantly, although the net cellular phenotype appears to be something of a dose-dependent effect (i.e., related to the *α*2 levels of the cells), the data shown in Figure 2(d)(ii) clearly show that *α*2 levels in the MP2-*α*2/CI cells removed from collagenI culture have returned to those observed in the MP2-*α*2/TC cells prior to the loss of invasion differences shown in Figure 3(a)(iv). This suggests that the effect on phenotype is more complex, and not solely due to the *α*2 expression level. Moreover, we did not identify any changes in *α*1, *α*3, *α*10, or *α*11 expression as a result of either stable *α*2 expression, or long-term interaction of the cells with collagenI, as noted in Figure 2(c)(i) and the Affymetrix results. Therefore, the phenotype observed is likely the direct result of *α*2 and downstream events reliant on continued *α*2 expression and/or engagement.

Mechanistically, loss of *α*2 by poorly differentiated PDAC cells would release their adhesive constraints within the collagen-rich desmoplasia of their primary site and may allow the cells to invade around, rather than through the collagen-rich matrix. This potentially unintentional capacity would mimic that of keratinocytes during their re-epithelialization of wound areas. Both resting and activated keratinocytes lack *α*v*β*3 integrin expression, and thus do not interact with fibrin or fibrinogen [25]. This provides a mechanism for promoting their migration and invasion around, rather than into, the fibrin clot, through the collagen-rich dermal wound margin and over fibronectin-rich granulation tissue. The antiadhesive nature of fibrin in this case provides the fundamental mechanism whereby the invading epidermis dissects the fibrin clot from the healing wound, resulting in an appropriately structured epidermis in place of the wound closure. In an analogous, albeit potentially disorganized manner, highly invasive, poorly differentiated, *α*2-negative PDAC cells may thus ignore the collagen matrix, and rather interact with other components such as laminin-5 (laminin-332), which has been shown to regulate migration of pancreatic tumor cells [26]. A similar mechanism has been demonstrated for fibroblast transmigration from collagenous stroma into the fibrin clot provisional matrix along fibronectin conduits [27]. Indeed, we observed altered adhesion (Figure 3(g)) and migration (not shown) on non-*α*2 matrix proteins in these cells, consistent with such a mechanism.

It is interesting to note that KLK-5, 6, and 7 expression is coordinately regulated by *α*2 in the PDAC system. In all cases, no other KLKs were affected, and all 3 KLKs were regulated in the same manner. Indeed, the PDAC lines examined demonstrate all or nothing expression of these KLKs, suggesting a coordinated regulation of KLK-5, 6 and 7 expression that may at least partly involve *α*2. KLK expression is cell- and tissue-dependent [28], and although KLK6 and KLK7 have been suggested to promote tumor cell invasion [14, 29, 30], loss of KLK5 has been observed in prostate [31], lung [32], breast [33], testicular [34], and renal cancer tissues [35] versus their normal counterparts, similar to our PDAC data presented herein. Based on the fact that KLK5 is expressed by more poorly-invasive PDAC cell lines, the fact that KLK blockade enhanced the invasion of KLK-positive cells to the exclusion of KLK-negative cells, and the fact that ectopic expression of KLK5 decreased invasion and collagenI migration of endogenously *α*2-positive PDAC cells, KLK5 appears to function in an anti-invasive manner in the PDAC system. Mechanistically, the negative effect of KLK5 on invasion likely reflects its negative influence on migration, as KLK blockade promoted enhanced collagenI migration by KLK5-positive cells (Figure 4(c)). 

Importantly, KLK5 was upregulated in MP2-*α*2Δ cells in comparison to MP2-*α*9*α*2 cells, suggesting that at least part of the invasion suppressor function of the *α*2-ectodomain is through modulation of KLK5 expression and that the *α*2 cytoplasmic domain retards invasion through regulation of other mediators. While the *α*2 cytoplasmic domain is dispensable for breast cancer cell adhesion to collagenI [36], spreading was compromised in that system. We did not observe such an effect, nor did the MP2-*α*2Δ cells demonstrate retarded collagenI migration (not shown). The *α*2-cytoplasmic domain is, however, required for EGF-induced chemotactic migration of mammary epithelial cells on collagenI [37]. Thus, the haptotactic response to collagenI measured in our system is likely independent of the growth-factor input that drives *α*2-cytoplamic domain-dependent chemotactic migration in breast cancer cells.

Although we show that *α*2 is a specific regulator of invasion in PDAC cells, there is evidence that the *α*2 integrin might function as a negative regulator of malignancy in other ways. Indeed, *α*2 signaling sensitizes MCF-7 and HepG2 cells deprived of matrix interactions to programmed cell death [38], suggesting that loss of *α*2 might be beneficial for a tumor cell that is disseminating into new tissue environments where matrix interactions may not be consistent. Mechanistically, ErbB2 and v-ras-mediated downregulation of *α*2-integrin expression has been shown to require the Sp1 transcription factor in human breast epithelial cells [39], an event that was tied to the disruption of tissue architecture observed in breast cancer. Previously we demonstrated the loss of syk tyrosine kinase associated with progression to poorly differentiated grade PDAC *in vitro* and *in situ* [12]. Syk has been shown to regulate Sp1 transcription factor activity in breast cancer cells; therefore, loss of syk may predispose breast epithelial cells to ErbB2-mediated downregulation of *α*2 integrin, resulting in a further step along the progression pathway. ErbB2 has also been implicated in PDAC malignancy [40]; therefore, it is possible that syk-dependent regulation of Sp1-mediated *α*2 integrin expression could serve as a switch in PDAC progression. Since over 6,000 transcripts were significantly affected by *α*2 in our system, the ramifications of such a switch could be dramatic.

Based on previous publications, the *α*2 integrin is clearly involved in mediating collagen interactions of well- to moderately differentiated PDAC cells, and indeed, has been suggested to mediate the malignancy of these cells [6]. However, while the *α*2 integrin does appear to mediate collagen adhesion and migration of some *α*2-positive PDAC cells, we provide evidence that reliance on the *α*2 integrin is progressively lost during PDAC “dedifferentiation”. Since *α*2-negative/independent cells demonstrate higher *in vitro* invasion and more pronounced distant dissemination in animals than their *α*2-dependent counterparts [10, 41], we propose that loss of *α*2 expression or utilization would promote the invasive phenotype in PDAC, at least partly through the regulation of KLK5 expression. Moreover, the *α*2-mediated regulation of gene products associated with invasion and dissemination indicates that *α*2 likely impacts tumor progression via both direct and indirect mechanisms. Further studies on the basis of these findings will investigate both avenues as well as the role of this integrin in regulating PDAC dissemination in an orthotopic animal model.

## Supplementary Material

Supplemental Materials included with this manuscript include a table detailing the relative differentiation state of the human cell lines used in this study, including the organ of origin of the original cells, the histological grade of the original primary tumor from which these cells were derived, the histological grade of the tumors produced by these cells in xenograft models, and a recent classification based on a detailed assessment of such criteria as molecular markers and the ability of the cells to organize into spheroids. Additionally, we provide a list of primers utilized in these studies, along with a reference to their use by other investigators. We also provide a table of representative gene changes observed by Affymetrix analysis of the MP2/mock, MP2/*α*2TC and MP2/*α*2CI cells, as well as a table detailing the Affymetrix-assessed expression of this subset of products in the MP2/*α*2∆ and MP2/*α*9*α*2 cells in reference to the MP2/*α*2CI cells. Finally, we provide detailed methodology for the Affymetrix Gene Array Analysis, including sample collection, microarray hybridization, data and ontology/pathway analysis, as well as statistical considerations. We further provide details of the image acquisition and analysis of the data presented in the text of the manuscript, and references for all supplemental material.

## Abbreviations

BSA: Bovine serum albumin
KLK: Kallikrein-related peptidase
MP2: MIAPaCa2
PDAC: Pancreatic ductal adenocarcinoma
SF: Serum-free.
