# Supplementary material for: α2 Integrin-Dependent Suppression of Pancreatic Adenocarcinoma Cell Invasion Involves Ectodomain Regulation of Kallikrein-Related Peptidase-5
Source: J Oncol. 2011 Dec 13;2011:365651. doi: 10.1155/2011/365651 (PMC3245846; doi:10.1155/2011/365651)
Supplement: Supplementary file 1 — Supplemental Materials included with this manuscript include a table detailing the relative differentiation state of the human cell lines used in this study, including the organ of origin of the original cells, the histological grade of the original primary tumor from which these cells were derived, the histological grade of the tumors produced by these cells in xenograft models, and a recent classification based on a detailed assessment of such criteria as molecular markers and the ability of the cells to organize into spheroids. Additionally, we provide a list of primers utilized in these studies, along with a reference to their use by other investigators. We also provide a table of representative gene changes observed by Affymetrix analysis of the MP2/mock, MP2/α2TC and MP2/α2CI cells, as well as a table detailing the Affymetrix-assessed expression of this subset of products in the MP2/α2∆ and MP2/α9α2 cells in reference to the MP2/α2CI cells. Finally, we provide detailed methodology for the Affymetrix Gene Array Analysis, including sample collection, microarray hybridization, data and ontology/pathway analysis, as well as statistical considerations. We further provide details of the image acquisition and analysis of the data presented in the text of the manuscript, and references for all supplemental material. [file 365651.f1.pdf]

SUPPLEMENTAL MATERIAL

Cell Line	Source of Cells	Histology/Grade of Primary Tumor	Histology/Grade of Transplanted Tumor	Recent Classification*
HPDE**	Normal Pancreas	N/A	N/A	Untransformed
CAPAN1	Liver Metastasis	PDAC/G1	G1	1
CAPAN2	Primary Tumor	PDAC/G1	G1	1
COLO357	Lymph Node Met	PDAC/G1-G2	G2/G3 (focally G1)	2
BxPC3	Primary Tumor	PDAC/G2	G2/G3	2
PANC1	Primary Tumor	PDAC/G3	G3	3
PT45P1	Primary Tumor	PDAC/G3	G3	3
MIAPaCa2	Primary Tumor	PDAC/G3	G3	3***

Supplementary Table S1. Relative differentiation of pancreatic ductal adenocarcinoma (PDAC) cells utilized in this study. This table shows the origin and grade of the original tumors from which the cell lines utilized in this study arose, as well as the grade of the tumors formed by these cells in mice. *Classification as proposed by Sipos et al. (2003). ** From Liu et al. (1998). ***The MIAPaCa2 cells were considered the least differentiated in this study due to the fact that they would not organize themselves into spheroids in culture. Table adapted from Sipos et al. (2003), with additional information from Liu et al. (1998) and Hotz et al. (2007).

Gene	Forward Primer<br>Reverse Primer	Source
GAPDH	5'-CCACCCATGGCAAATTCCATGGCA-3'<br>5'-TCTAGACGGCAGGTCAGGTCCACC-3'	Stratagene
Integrin α1	5'-TGCCAGTGAGATTTCAGAGACC-3'<br>5'- GTGATTTCCTGTGTTTTCGTCG-3'	Rogojina et al, 2003
Integrin α2	5'-AACTCTTTGGATTTGCGTGTG-3'<br>5'-TGGCAGTCTCAGAATAGGCTTC-3'	Rogojina et al, 2003
Integrin α3	5'-ACTGTGAAGGCACGAGTGTG-3'<br>5'-TGCTGGTTCGGAGGAATAG-3'	Rogojina et al, 2003
Integrin β1	5'-CAAAGGAACAGCAGAGAAGC-3'<br>5'-ATTGAGTAAGACAGGTCCATAAGG-3'	Rogojina et al, 2003
KLK-5	5'-GAGCTGGGGCCGGGGAAGAC-3'<br>5'-TGGGCCGGGCACAAGGGTAA-3'	Harvey et al., 2000
KLK-6	5'-GGAGGAATTCAGCAGGAGCGGCCATG-3'<br>5'-TGTCTCGAGTCAGGGTCACTTGGCCTG-3'	Harvey et al., 2000
KLK-7	5'-GCCCAGGGTGACAAGATTATT-3'<br>5'-GTACCTCTGCACACCAACGG-3'	Harvey et al., 2000
KLK-5 (CDS)	5'-GCtctagaCCGATGGTGGCCCGTCCTTG-3'<br>5'-CGtctagaGGGGATGCCGGTGTGCTGAG-3'	N/A
KLK-6 (CDS)	5'-GCtctagaCAAGAATCCCCGGAGGCCCG-3'<br>5'-CGtctagaGGGGTGGTAGGTCGGGAGGT-3'	N/A
α2Δcyto/XbaI reverse	5'-TTGCAATTTTATGGTAGTCTAGAGG-3'	N/A

Supplementary Table S2. PCR Primers and Sources

Gene Title	Detection Mock/α2TC/α2CI	LogFoldChange Mock vs. α2TC	LogFoldChange Mock vs. α2CI
Kallikrein-related peptidase 6	A/P/P	3.4	6.7
Kallikrein-related peptidase 5	A/P/P	0.1	2.5
Kallikrein-related peptidase 7	A/A/P	0.1	1.7
Rho GTPase-activating protein 15	A/P/P	5.0	4.7
Neural precursor, developmentally down-regulated 4-like	A/P/P	1.4	2.1
ATPase, class I, type 8B, member 1	A/P/P	1.9	1.7
IQ motif containing GTPase activating protein 2	A/P/P	1.6	1.6
pentraxin-related gene, rapidly induced by IL-1 β	P/P/P	3.0	2.6
nuclear receptor subfamily 5, group A, member 2	P/P/P	2.9	2.0
receptor tyrosine kinase-like orphan receptor 1	P/P/P	1.5	1.5
Aldolase C, fructose-bisphosphate	P/P/A	-1.0	-1.0
cAMP responsive element binding protein 3-like 1	M/A/A	-1.4	-1.3
Angiotensin I converting enzyme 1	P/A/A	-1.4	-1.4
Hexokinase domain containing 1	P/A/A	-2.5	-2.5
S100 calcium binding protein A3	P/P/P	-2.2	-1.8
Chemokine (C-X-C motif) ligand 2	P/P/P	-1.6	-2.2
Chitinase 3-like 2	P/A/A	-2.2	-2.3
Cathepsin C	P/P/P	-3.7	-4.3
Bone marrow stromal cell antigen 2	P/A/A	-5.8	-5.9

Supplementary Table S3. Affymetrix array Summary I (Mock/α2TC/α2CI). Presented is a subset of some of the most highly altered genes. The full set of Affymetrix data is available in the GEO repository via Accession #GSE18277 (http://www.ncbi.nlm.nih.gov/geo/query/acc.cgi?token=zreftooauqiuupq&acc=GSE18277). P, present; M, marginal; A, absent.

Gene Title	Detection α2CI/α2Δ/α9α2	LogFoldChange α2CI vs. α2Δ	LogFoldChange α2CI vs. α9α2
Kallikrein-related peptidase 6	P/P/A	-2.9	7.2
Kallikrein-related peptidase 5	P/P/A	-3.0	4.7
Kallikrein-related peptidase 7	P/P/A	0.4	1.5
Rho GTPase-activating protein 15	P/A/A	6.9	3.4
Neural precursor, developmentally down-regulated 4-like	P/A/A	3.3	3.3
ATPase, class I, type 8B, member 1	P/A/P	2.5	1.8
IQ motif containing GTPase activating protein 2	P/A/A	4.1	4.1
pentraxin-related gene, rapidly induced by IL-1 β	P/P/A	2.9	7.9
nuclear receptor subfamily 5, group A, member 2	P/A/A	5.0	5.0
receptor tyrosine kinase-like orphan receptor 1	P/A/A	2.6	2.6
Aldolase C, fructose-bisphosphate	A/P/P	0.0	0.0
cAMP responsive element binding protein 3-like 1	A/M/P	-1.4	-1.0
Angiotensin I converting enzyme 1	A/P/P	-0.3	-1.4
Hexokinase domain containing 1	A/A/P	0.0	-0.4
S100 calcium binding protein A3	P/P/A	-0.5	1.0
Chemokine (C-X-C motif) ligand 2	P/P/P	-1.5	1.1
Chitinase 3-like 2	A/P/P	-4.9	-2.7
Cathepsin C	P/P/P	1.5	1.9
Bone marrow stromal cell antigen 2	A/A/P	-0.1	-0.5

Supplementary Table S4. Affymetrix array Summary II (α2CI/α2Δ/α9α2). Presented is the subset of genes shown in Supplemental Table S3, with comparison of MP2-α2Δ and MP2-α9α2 to MP2-α2CI. The full set of Affymetrix data is available in the GEO repository via Accession #GSE18277 (http://www.ncbi.nlm.nih.gov/geo/query/acc.cgi?token=zreftooauqiuupq&acc=GSE18277). P, present; M, marginal; A, absent.

SUPPLEMENTAL EXPERIMENTAL PROCEDURES

Affymetrix Gene Array Analysis

Performed essentially as described previously (Goodison et al., 2007).

Sample collection and preparation of labeled RNA: Total RNA was extracted from each of the cell line samples and prepared for hybridization according to the GeneChip Expression Analysis Technical Manual (Affymetrix, Santa Clara, CA, USA). Briefly, samples were homogenized in lysis buffer and the RNA extracted (RNeasy Mini kit; Qiagen, Inc., Valencia, CA, USA). The quality of each RNA sample was assessed after running 200-ng aliquots through a microchannel RNA analysis chip on a bioanalyzer (RNA 6000 Nano Chip; Agilent Technologies, Palo Alto, CA, USA) and evaluating the relative amounts of 28S and 18S ribosomal peaks. A 5μg aliquot of RNA was used as a template for complementary DNA (cDNA) synthesis (Superscript Choice System kit; Invitrogen Life Technologies, Gaithersburg, MD, USA). First-strand synthesis was primed with a T7-(dT) 24mer oligonucleotide primer containing a T7 RNA polymerase promoter sequence on the 5' end (Genset Oligos, La Jolla, CA, USA). Second-strand products were cleaned (GeneChip Sample Cleanup Module; Affymetrix) and used as a template for in vitro transcription (IVT) with biotin-labeled nucleotides (Bioarray High Yield RNA Transcript Labeling Kit; Enzo Diagnostics, Farmingdale, NY, USA). The copy RNA (cRNA) product was cleaned with the cleanup module and a 20μg aliquot was heated at 94°C for 35 min in fragmentation buffer provided with the cleanup module (Affymetrix).

Microarray hybridization: Fifteen micrograms of adjusted cRNA from each sample was hybridized for 16 h at 45 °C to an Affymetrix U133 Plus 2.0 GeneChip array, which offers coverage of 47,000 transcripts. After hybridization, each array was stained with a streptavidin-phycoerythrin conjugate (Molecular Probes, Eugene, OR, USA), washed and visualized with a microarray scanner (Genearray Scanner; Agilent Technologies, Santa Clara, CA, USA). Images were inspected visually for hybridization artifacts. In addition, quality assessment metrics were generated for each scanned image and evaluated based on empiric data from previous hybridizations and the signal intensity of internal standards present in the hybridization cocktail.

Generation of expression values: Microarray Suite, version 5 (Affymetrix), was used to generate .cel files, and a computer program (Probe Profiler, ver. 1.3.11; Corimbia Inc., Berkeley, CA, USA) developed specifically for the GeneChip system (Affymetrix) was used to convert intensity data into quantitative estimates of gene expression for each probe set. A probability statistic was generated for each probe set (gene). The probability is associated with the null hypothesis that the expression level of the probe set is equal to zero (background). Genes not significantly expressed above background in at least two samples ($P<0.05$) were considered absent.

Gene expression data analysis: Statistical tests were performed using BioConductor statistical software (http://www.bioconductor.org/) which is an open source and open development software project for analysis of microarray and other high-throughput data based primarily on the R programming language (Gentlemen, et al., 2004). The raw data were normalized by Robust Multichip Analysis (RMA) approach implemented in Affy package (Bolstad, et al., 2003). The fold-change was then computed based on the normalized data. Significant p-value was computed by a statistic test based on a probe level analysis using affyPLM package (Bolstad, et al., 2005). The p-values were further adjusted using the Benjamini and Hochberg method (Benjamini and Hochbert, 1995). Differentially expressed genes were than ranked by the adjusted p-values, and genes with the p-value of less than 0.05 were considered as differentially expressed genes at a statistically significant level.

Gene Ontology and Pathway analysis: Gene Ontology annotations were obtained from Affymetrix. Biological network relationships among significantly regulated genes were explored using KEGG and GenMapp pathways using AnalyzeIt Tools.

Image Acquisition and Manipulation

Images of ethidium bromide-stained agarose gels were captured with Quantity One software on a Bio-Rad Gel Doc XR using the appropriate filter and transmitted UV light. Chemiluminescence-exposed films and printouts of agarose gels were scanned on an EpsonPerfection 4490Photo flatbed

scanner. Band intensities of unadjusted images were densitometrically analyzed using NIH Image 1.61, corrected for appropriate loading controls and compared to the indicated baseline comparison band, which was defined as 1.0. Brightfield images of immunohistochemically stained patient samples were acquired with a Nikon TE600 microscope with a Model 3.2.0 CCD camera (Diagnostic Instruments, Sterling Heights, MI, USA) using Spot™ software. Images were imported into Adobe Photoshop for removal of unused levels and cropping. Minimal alterations to brightness and contrast were employed for a subset of images, to improve the visual nature of the image. Non-linear adjustments were not employed. Final images were compiled in Adobe InDesign, rasterized and converted to jpeg format at a minimum of 300 dpi.

SUPPLEMENTARY REFERENCES

Benjamini Y and Hochberg Y. (1995) Controlling the False Discovery Rate: a Practical and Powerful Approach to Multiple testing. *J Royal Stat Soc B* **57,** 289-300.

Bolstad BM, Irizarry RA, Astrand M, Speed TP. (2003) A comparison of normalization methods for high density oligonucleotide array data based on variance and bias. *Bioinformatics* **19,** 185-19.

Bolstad BM, Collin F, Brettschneider J, Simpson K, Scope L, Irizarry RA, and Speed TP. (2005) Quality Assessment of Affymetrix GeneChip Data**.** In Bioinformatics and Computational Biology Solutions Using R and Bioconductor. Edited by: Gentleman R, Carey V, Huber W, Irizarry R, Dutoit S. Heidelberg: Springer.

Gentleman RC, Carey VJ, Bates DM, Bolstad B, Dettling M, Dudoit S, et al. (2004) Bioconductor: open software development for computational biology and bioinformatics. *Genome Biol* **5,** R80.

Goodison S, Nakamura K, Iczkowski KA, Anai S, Boehlein SK, Rosser CJ. (2007) Exogenous mycoplasmal p37 protein alters gene expression, growth and morphology of prostate cancer cells. *Cytogenet Genome Res* **118,** 204-13

Harvey TJ, Hooper JD, Myers SA, Stephenson SA, Ashworth LK, Clements JA. (2000) Tissue-specific expression patterns and fine mapping of the human kallikrein (KLK) locus on proximal 19q13.4. *J Biol Chem* **275,** 37397-37406.

Liu, N, Furukawa, T, Kobari, M, Tsao, M-S. (1998) Comparative phenotypic studies of duct epithelial cell lines derived from normal human pancreas and pancreatic carcinoma. *Am J Pathology* **153,** 263-269.

Rogojina AT, Orr WE, Song BK, Geisert EE Jr. (2003) Comparing the use of Affymetrix to spotted oligonucleotide microarrays using two retinal pigment epithelium cell lines. *Mol Vis* **9,** 482-496.

Sipos B, Moser S, Kalthoff H, Torok V, Lohr M, Kloppel G. (2003) A comprehensive characterization of pancreatic ductal carcinoma cell lines: towards the establishment of an in vitro research platform. *Virchows Arch* **442,** 444-452.
